# Häufigkeit und Barrieren bei Patientenverfügung und Vorsorgevollmacht: Erkenntnisse aus einer prospektiven Observationsstudie im palliativmedizinischen Dienst

**DOI:** 10.1007/s00063-024-01149-5

**Published:** 2024-05-13

**Authors:** Benedict Mathias Breen, Claudia Flohr, Heike Wendt, Katharina Chalk, Ulrike Haase, Christiane Hartog, Sascha Tafelski

**Affiliations:** 1 Klinik für Anästhesiologie und Intensivmedizin, Campus Charité Mitte und Campus Virchow-Klinikum, Charité – Universitätsmedizin Berlin, Charité – Universitätsmedizin Berlin, Corporate Member der Freien Universität Berlin und Humboldt-Universität zu Berlin, Charitéplatz 1, 10117 Berlin, Deutschland; 2 Zentrales Pflegecenter (ZPC), Palliativkonsildienst, Charité – Universitätsmedizin Berlin, Gliedkörperschaft der Freien Universität Berlin, der Humboldt Universität zu Berlin und dem Berlin Institute of Health, Charitéplatz 1, 10117 Berlin, Deutschland; 3 Zentrum für Schmerzmedizin, Ostseestraße 107, 10409 Berlin, Deutschland; 4 Klinik Bavaria Kreischa, 01731 Kreischa, Deutschland

**Keywords:** Patientenverfügung, Vorsorgevollmacht, Autonomie, Vertretungsregelung, Palliativmedizin, Advance directive, Health care proxy, Power of attorney, Patient autonomy, Palliative car

## Abstract

**Hintergrund und Fragestellung:**

Vorsorgevollmacht und Patientenverfügung sind Instrumente zur Stärkung der Patientenautonomie. Palliativmedizinische Dienste bieten entsprechende Beratung für Patient:innen und Angehörige an. Gegenstand der vorliegenden Studie ist es, den Beratungsbedarf von lebensbedrohlich erkrankten Patient:innen zu erfassen.

**Studiendesign und Untersuchungsmethode:**

Diese prospektive Beobachtungsstudie erfasste stationäre Patient:innen mit einer palliativmedizinischen Mitbehandlung. Es wurden patientenbezogene Faktoren ermittelt, die mit dem Vorliegen von Vorsorgevollmacht oder Patientenverfügung assoziiert waren. Zusätzlich erfolgten Fokusgruppeninterviews mit Mitgliedern des Konsildiensts zur Ermittlung von Barrieren zur Erstellung von Vorsorgeverfügungen.

**Ergebnisse:**

Insgesamt wurden 294 Patient:innen eines palliativmedizinischen Diensts mit einem medianen Alter von 67 Jahren eingeschlossen, davon 69 (23 %) intensivmedizinisch behandelte Patient:innen. Insgesamt 98 (33 %) Patient:innen hatten eine Patientenverfügung, 133 (45 %) eine Vorsorgevollmacht. Es verstarben 52 Patient:innen (17,7 %) im Krankenhaus. Lediglich das Alter sowie der Familienstand waren mit dem Vorhandensein von Verfügungen assoziiert. Fokusgruppeninterviews erarbeiteten folgende Barrieren: Informationsdefizite, Sorgen vor einem Therapieabbruch, Autonomieverlust sowie den Wunsch, Belastung der Familie bzw. Patientenvertreter zu vermeiden.

**Diskussion:**

Es zeigt sich, dass auch in dieser schwer erkrankten Population die Mehrheit keine Vorausverfügung trifft. Um Hürden abzubauen, ist eine verbesserte Aufklärung und Beratung über Vorausverfügungen notwendig, insbesondere in spezifischen Situationen der eigenen Urteilsunfähigkeit.

## Abstract (en)

**Background:**

The advance directive and lasting power of attorney are instruments to strengthen patients’ autonomy. A hospital-based palliative care consultation service can advise patients and family members about these instruments. This study investigates the need for such consultation among patients with life-limiting illness.

**Methods:**

This prospective observational study on intensive and non-intensive care units includes patients with a request for palliative care consultation. Patient-related factors were evaluated for their possible association with the presence or absence of advance directives or power of attorney. In addition, focus group interviews with members of the palliative care consultation team were carried out to identify barriers which prevent patients from drawing up such documents.

**Results:**

A total of 241 oncological and 53 non-oncological patients were included with a median age of 67 years; 69 (23%) patients were treated in the intensive care unit (ICU). Overall, 98 (33%) patients had advance directives, and 133 (45%) had determined a legal health care proxy in advance. A total of 52 patients died in hospital (17.7%). Only age and relationship status were associated with directives. In interviews, the following barriers were identified: information deficit, concern regarding discontinuation of treatment, loss of autonomy and wish to avoid a burden for the family.

**Conclusion:**

The majority in this severely ill patient population lack advance directives. In order to remove barriers, more effective information and counseling is required about such directives. In particular, guidance should include potential clinical situations in which such directives are potentially beneficial.

## Hintergrund und Fragestellung

Mit einer *Patientenverfügung* können Menschen ihrer individuellen Autonomie auch im Fall einer kritischen Erkrankungssituation Ausdruck verleihen. Patient:innen können für den Fall, dass sie selbst nicht mehr in der Lage sind, Entscheidungen zu treffen, ihren Willen vorausschauend dokumentieren [5, 6]. Mit einer *Vorsorgevollmacht* definieren Menschen diejenige Person, die im Fall eigener Entscheidungsunfähigkeit Entscheidungen in medizinischen Angelegenheiten übernehmen darf. Die Vorsorgevollmacht ermächtigt eine Person, den Willen von Patient:innen in Gesundheitsfragen zu vertreten, auch wenn eine schriftliche Patientenverfügung nicht vorliegt.

Mit diesen Verfügungen hat der Gesetzgeber die Autonomie von Patient:innen umfassend gestärkt. Ergänzend wurde zum Januar 2023 mit dem *Notvertretungsrecht für Ehegatten* eine Neuregelung umgesetzt, die eine zeitlich begrenzte, nicht übertragbare Berechtigung für den Fall ermöglicht, in dem keine Vorsorgevollmacht besteht [3]. Aufgrund der Fortschritte in der intensivmedizinischen Therapie und der Zunahme an älteren Patient:innen mit komplexeren Erkrankungen müssen immer häufiger Behandlungsentscheidungen bei Patient:innen getroffen werden, die nicht in der Lage sind, ihren Willen zu äußern. Es wäre wünschenswert, wenn Patient:innen mit einer hohen Krankheitslast oder mit einem erhöhten Risiko zu versterben Verfügungen für den Fall der Entscheidungsunfähigkeit vorbereiten würden, damit medizinische Entscheidungen im Einklang mit den Wünschen der Patient:innen getroffen werden können.

Obwohl der Bekanntheitsgrad von Vorsorgevollmacht und Patientenverfügung in der allgemeinen Bevölkerung inzwischen sehr hoch ist (zwischen 80–100 %) [4], fällt auf, dass nur 43 % der Befragten ein solches Dokument tatsächlich erstellt haben. In einer weiteren Untersuchung im Jahr 2017–2018 hatten insgesamt 29 % der Patient:innen, die in einem kommunalen Krankenhaus verstarben, eine Vorsorgevollmacht, eine Patientenverfügung lag hier nur bei 27 % vor [12]. In einer deutschen multizentrischen Studie mit über 1000 intensivmedizinischen Patient:innen, bei denen Behandlungsentscheidungen am Lebensende getroffen wurden, hatten nur 44 % einen Bevollmächtigten und nur 27 % besaßen eine Patientenverfügung; die behandelnden intensivmedizinischen Ärzt:innen konnten in bis zu 50 % der Fälle keine hinreichenden Informationen über Patientenpräferenzen zu Behandlungszielen erhalten [2].

Eine frühzeitige palliativmedizinische Mitbehandlung von onkologischen Patient:innen führte in einer randomisierten kontrollierten Studien zu einer signifikanten Verbesserung der Lebensqualität [14]. Eine entsprechende Begleitung wird zunehmend auch für Patient:innen ohne Tumordiagnose angeboten [7]. Hierzu gehören Patient:innen beispielsweise mit schweren Lungenerkrankungen (z. B. COPD), schweren chronischen Organversagen (z. B. Leberinsuffizienz), terminaler Herzinsuffizienz oder progredienten neurologischen Erkrankungen (z. B. Demenz, Motoneuronerkrankungen wie ALS). Insbesondere in dieser Population sind akute, organbezogene Exazerbationen oft Indikation für eine intensivmedizinische Behandlung. Die therapeutischen Präferenzen und Behandlungsziele von Patient:innen sind von zentraler Bedeutung für die Indikationsstellung der weiteren Therapieeskalation oder Therapiebegrenzung. Entsprechend wichtig sind in dieser Situation der dokumentierte Patientenwillen und die Kenntnis von vorsorgeberechtigten Zugehörigen als Ansprechpartner in der weiteren Krankenhausbehandlung.

Die Klinik für Anästhesiologie und Intensivmedizin der Charité – Universitätsmedizin Berlin hat seit 2013 einen palliativmedizinischen Konsildienst etabliert, der für intensivmedizinische wie auch normalstationäre Patient:innen hinzugezogen werden kann. Er besteht aus einem multiprofessionellen ärztlichen und pflegerischen Team und arbeitet eng mit Sozialdienst, Physiotherapie, Psychoonkologie sowie dem Behandlungsteam der Normal- und Intensivstation zusammen.

Vor diesem Hintergrund wurde im Rahmen einer prospektiven Observationsstudie untersucht, welche Faktoren mit der Erstellung von Vorausverfügungen bei stationären Patient:innen assoziiert sind, bei denen der palliativmedizinische Konsildienst hinzugezogen wurde.

## Studiendesign und Untersuchungsmethoden

Es erfolgte eine prospektive Beobachtungsstudie an der Charité – Universitätsmedizin Berlin, Campus Charité Mitte. Die Studie wurde von der Ethikkommission der Charité positiv beraten (EA4/102/20) und durch den behördlichen Datenschutz geprüft.

Im Studienzeitraum dieser Untersuchung vom 01.06.2020 bis zum 30.04.2021 wurden alle Patient:innen evaluiert, bei denen eine palliativmedizinische Mitbehandlung angefordert wurde. Patient:innen, bei denen das palliativmedizinische Basisassessment nicht durchgeführt werden konnte oder nicht vorlag, wurden ausgeschlossen. Bei Wiederaufnahme von Patient:innen erfolgte keine erneute Dokumentation in der Studiendatenbank. Die Datenerfassung erfolgte während des palliativmedizinischen Erstgesprächs in einem multidisziplinären Behandlungsteam zusammen mit spezifisch ausgebildeten Pflegefachkräften mit Zusatzweiterbildung in *„palliative care“*. Die Daten wurden mittels „minimal documentation system“ (MIDOS) ermittelt, einem Selbsteinschätzungsfragebogen, der zur Symptomeinschätzung von Palliativpatienten validiert [9] und adaptiert wurde [13]. Die hier abgefragten Symptome quantifizieren die Symptomkomplexe Übelkeit, Erbrechen, Verstopfung, Schwäche, Appetitmangel, Müdigkeit, Erschöpfung, Schlafstörung, Wundprobleme, Atemnot, Lymphödem, Hilfsbedarf bei täglichen Aktivitäten, Depression, Antriebsmangel, Angst, Verwirrtheit, Desorientiertheit sowie Versorgungsprobleme oder eine Überforderung der Familie. Der von der Eastern Cooperative Oncology Group (ECOG) vorgeschlagene ECOG-Performance-Status zur Erfassung der Alltagskompetenz und Mobilität wurde als 6‑stufige Graduierung von 0–5 erhoben [8]. Weitere Faktoren waren der BMI, Ernährungsstatus mit quantifiziertem Gewichtsverlust sowie die Disstressintensität [16]. Bei Letzterem werteten die Patient:innen ihre Lebensqualität als *gar nicht belastet* (0) bis *extrem belastet* (10). Ein Schmerzassessment mit Medikationsanamnese wurde durchgeführt. Hierbei erfolgte eine Zuordnung der Schmerzqualität sowie Schmerzintensität anhand der numerischen Rating-Skala (NRS) zwischen 0 und 10.

Im Rahmen des Assessment wurden Patient:innen zum Vorliegen einer Vorsorgevollmacht und einer Patientenverfügung befragt. Bei Vorliegen einer Patientenverfügung wurde zudem differenziert, ob diese auch Regelungen zur Vorsorgevollmacht beinhaltete.

Zur Erfassung von Barrieren wurden *3 Fokusgruppeninterviews* im Bereich des palliativmedizinischen Konsildiensts mit ärztlichen und pflegerischen Mitarbeiter:innen geführt. Es wurde hierbei spezifisch nach Hindernissen gefragt, die während der palliativen Mitbehandlung durch Patient:innen oder An- und Zugehörigen geäußert wurden. Entsprechende Antworten wurden zusammengetragen, durch die Autoren (ST, HW, KC) kategorisiert und in einer Tabelle zusammengefasst. Ziel war es, die Barrieren zu ermitteln, die die Patient:innen von der Erstellung der Verfügungsinstrumente abhielten.

## Statistische Analyse

Die Datenanalyse erfolgte mittels SPSS Version 28.0 (IBM, New York, USA). Die deskriptive Datenanalyse wurde entsprechend dem Skalenniveau und nach Prüfung auf Normalverteilung dargestellt und hierfür entsprechend Mittelwerte mit Standardabweichung oder Mediane mit 25 %-/75 %-Quartilen gewählt. Für die univariate Analyse auf statistische Signifikanz der Daten wurde entsprechend der Verteilung der Mann-Whitney-U-Test sowie der exakte Test nach Fisher angewendet. Das statistische Signifikanzniveau wurde aufgrund des beobachtenden Charakters dieser Untersuchung mit einem *p* ≤ 0,05 definiert. In einer logistischen Regressionsanalyse erfolgte eine explorative Analyse der Barrieren für das Vorliegen einer Patientenverfügung und Vorsorgevollmacht. Die von den Konsildienstmitarbeiter:innen erfassten Barrieren wurden deskriptiv dargestellt.

## Ergebnisse

**Abb. 1:**
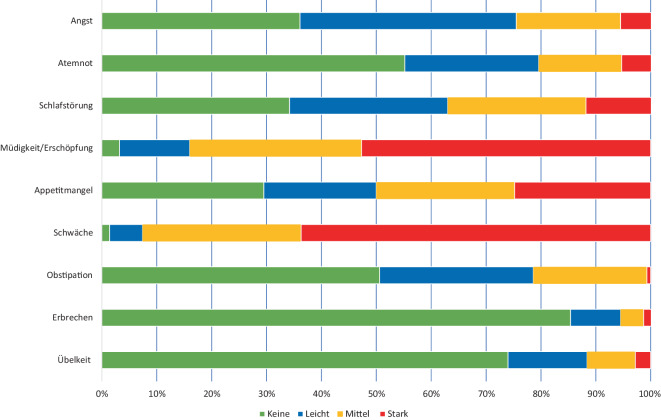
Übersicht über die Symptombelastung von Patient:innen im palliativmedizinischen Assessment

Insgesamt wurden im Studienzeitraum *N* = 555 Patient:innen durch den Konsildienst evaluiert. Nach Ausschluss von Wiedervorstellungen und von Patient:innen mit unvollständigem Basisassessment konnten 294 Patient:innen in die Analyse eingeschlossen werden. Das mediane Alter der Studienpopulation betrug 67 Jahre, 44 % waren weiblich und 23 % wurden primär auf einer Intensivstation evaluiert (siehe Tab. 1). Insgesamt 241 (82 %) Patient:innen wurden aufgrund einer führend onkologischen Erkrankung und 53 (18 %) Patient:innen aufgrund einer führend nichtonkologischen Erkrankung behandelt. Insgesamt 52 Patient:innen (17,7 %) verstarben während des beobachteten Krankenhausaufenthalts. Eine völlige oder teilweise Pflegebedürftigkeit nach ECOG lag bei 231 (82,2 %) der Patient:innen vor. Mittels Disstressthermometer gaben die Patient:innen eine deutliche Einschränkung der Lebensqualität mit im Median 7 Punkten an. Bei rund einem Drittel der Patient:innen bestand zudem eine mittlere bis starke Überforderung der Familie (mittel 29 %, stark 11,5 %). Über depressive Symptome und Antriebsmangel berichteten mehr als die Hälfte der Patient:innen (mittel 15 %, stark 8,8 %). Die weitere Symptomlast der Patient:innen wird in Abb. 1 dargestellt. Die höchste Symptomlast wurde bei Müdigkeit/Erschöpfung und bei Schwäche genannt.

**Tab. 1:** Charakteristika der Studienpopulation. Angaben in Median mit Quartilen oder absolute und relative Anzahl (*N*, %)

**Patient:innen**	***n*** **=** **294**
*Alter – Jahre*	67 (56–77)
*BMI – kg/m*^*2*^	24,2 (20,9–27,9)
*Weiblich*	129 (43,9 %)
*Onkologische Patient:innen*	241 (82 %)
*Nichtonkologische Patient:innen*	53 (18 %)
*Intensivmedizinische Patient:innen*	69 (23,4 %)
*Familienstand – ledig*	117 (42,6 %)
*Religiöse Zugehörigkeit*	82 (31,5 %)
*ECOG-Performance-Status*^*a*^	
0	0 (0 %)
1	5 (1,7 %)
2	47 (16 %)
3	83 (31,7 %)
4	148 (50,5 %)
5	–
*Disstresstermometer* (von 0, „gar nicht belastet“, bis 10, „extrem belastet“)	7 (6–8)
**Patient:innen ohne Familie, *n* (%)**	
*Depression/Antriebsmangel*	
Keine	115 (42,3 %)
Leicht	91 (33,5 %)
Mittel	42 (15,4 %)
Stark	24 (8,8 %)
*Überforderung der Familie*	
Ohne Familie	34 (11,5 %)
Familie nicht überfordert	24 (8,2)
Leicht überfordert	57 (19,4 %)
Mittelgradig überfordert	93 (31,6 %)
Stark überfordert	86 (29,3 %)
*Vorsorgevollmacht vorhanden*	133 (45 %)
*Patientenverfügung vorhanden*	98 (33 %)
*Teilnehmer in Fokusgruppen*	
Mitglieder im Konsildienst	19
Davon Weiblich	12
Davon Ärzte	8
Davon Pflegekräfte	11

^a^*ECOG* Eastern Cooperative Oncology Group, von 0 (uneingeschränkte Aktivität) bis 5 (völlige Pflegebedürftigkeit)

## Vorsorgevollmacht und Patientenverfügung

Insgesamt 98 Patient:innen gaben an, dass sie bereits eine Patientenverfügung erstellt hatten (33 %) und 133 Patient:innen berichteten über eine dokumentierte Vorsorgevollmacht (45 %). Es bestand kein Unterschied in der Häufigkeit zwischen onkologischen und nichtonkologischen Patient:innen (*p* = 0,418 und *p* = 0,315). Insgesamt hatten von *n* = 98 Patient:innen mit einer Patientenverfügung nur *n* = 8 keine Regelung zur Vorsorgevollmacht mit eingeschlossen (8,2 %).

Die Tab. 2 zeigt die Assoziation zwischen Charakteristika von Patient:innen und dem Fehlen einer Patientenverfügung. Es zeigt sich, dass die Wahrscheinlichkeit, eine Patientenverfügung zu besitzen, mit dem Alter zunahm. Weder Geschlecht, Angst, Depressivität, aktueller Disstress oder der Familienstand waren mit dem Vorhandensein einer Patientenverfügung assoziiert. Auch das Versterben von Patient:innen innerhalb des aktuellen Aufenthalts war nicht mit dem Vorhandensein einer Patientenverfügung zu Behandlungsbeginn assoziiert.

**Tab. 2:** Univariate logistische Regressionsanalysen zur Assoziation zwischen Kovariablen und dem Fehlen einer Vorsorgevollmacht und Patientenverfügung

Variable	Vorsorgevollmacht		Patientenverfügung	
	OR (95 %-KI)	*p*-Wert	OR (95 %-KI)	*p*-Wert
Alter	0,962 (0,945–0,980)	*<* *0,001*	0,957 (0,938–0,976)	*<* *0,001*
Geschlecht (weiblich vs. männlich)	1,079 (0,679–1,714)	*0,749*	0,781 (0,479–1,271)	*0,319*
Angst	0,820 (0,623–1,080)	*0,158*	0,853 (0,642–1,134)	*0,275*
Depressivität	0,808 (0,629–1,038)	*0,095*	0,974 (0,751–1,265)	*0,846*
Überforderung der Familie	0,895 (0,703–1,140)	*0,369*	0,971 (0,756–1,247)	*0,819*
Familienstand ledig	1,810 (1,111–2,950)	*0,017*	1,322 (0,792–2,206)	*0,285*
Religiosität	1,551 (0,905–2,658)	*0,111*	1,580 (0,885–2,823)	*0,122*
Disstressthermometer (0–10)	0,908 (0,790–1,043)	*0,173*	0,935 (0,808–1,081)	*0,363*
Verstorben	0,598 (0,327–1,093)	*0,095*	1,036 (0,547–1,960)	*0,914*

Die Tabelle zeigt ebenfalls die Faktoren, die mit dem Fehlen einer Vorsorgevollmacht assoziiert waren. Sowohl das Lebensalter als auch der Familienstand (Leben in einer Partnerschaft) zeigten eine signifikante Assoziation. Weder Geschlecht, Angst, Depressivität oder der erlebte Disstress waren mit dem Vorliegen einer Vorsorgevollmacht assoziiert.

## Qualitative Analyse von Barrieren zur Vorsorgevollmacht und Patientenverfügung

Die Tab. 3 zeigt eine Übersicht über häufig genannte Barrieren. Zwei Kategorien betreffen ein Informationsdefizit:

- Patient:innen waren nicht informiert über mögliche Situationen, in denen Vertreterentscheidungen benötigt würden wie z. B. die Anlage eines Tracheostomas oder einer PEG-Sonde oder bei der Frage der Wiederbelebung nach Herzstillstand. Insbesondere war der Nutzen einer Vorausverfügung häufig unklar.
- Patient:innen wussten nicht, dass eine notarielle Beglaubigung nicht benötigt wird.

**Tab. 3:** Häufig genannte Barrieren für die Erstellung einer Vorsorgevollmacht oder Patientenverfügung

Barrieren
Nutzen der Verfügung nicht bekannt
Möglichkeit der Formulierung ohne notarielle Begleitung nicht bekannt
Sorge vor Limitierung potenzieller Behandlungsangebote
Sorge vor Entmündigung bei erhaltener Einsichtsfähigkeit (Autonomieverlust)
Sorge vor der Bürde der Verantwortung beim Bevollmächtigten bzw. Zugehörigen
Religiöse oder spirituelle Überzeugungen

Zwei weitere Kategorien betreffen eine mögliche Problematik, die durch eine Verfügung entstehen könnte: Limitierung möglicher Behandlungsangebote und möglicher Verlust der Autonomie. Patient:innen äußerten die Sorge, dass eine Therapie zu früh eingeschränkt werden könnte oder dass der Bevollmächtigte entgegen der Patienteninteressen handeln könnte. Ebenfalls vorgetragen wurden auch religiöse oder spirituelle Überzeugungen, die der Formulierung von Therapiebegrenzungen entgegenstehen könnten.

## Diskussion

In dieser Untersuchung von Patient:innen, die auf Intensiv- und Normalstationen einer Universitätsklinik zur palliativmedizinischen Mitversorgung vorgestellt wurden, zeigte sich, dass eine Patientenverfügung oder eine Vorsorgevollmacht nur bei 33 % der Patient:innen vorhanden war. Nur das zunehmende Lebensalter war mit dem Vorhandensein einer Verfügung oder Vollmacht assoziiert, nicht dagegen psychologische Faktoren, wie Angst oder Depressivität, oder die Überforderung der Familie und auch nicht das Versterben im Krankenhaus. Jeder zweite Untersuchte besaß zum Untersuchungszeitpunkt keine Vorsorgedokumente. Lediglich das Vorhandensein eines Partners war damit assoziiert, dass Patient:innen eine Vorsorgevollmacht erstellt hatten. Aus Sicht der im Konsildienst tätigen Mitarbeiter:innen hinderten vor allem nichtmedizinische Gründe das Erstellen einer Verfügung, z. B. das Nichtwissen darüber, wofür bzw. in welcher Situation eine Verfügung nützlich sein könnte und dass eine Verfügung auch ohne notarielle Beglaubigung Gültigkeit besitzt. Weit verbreitet war auch die Sorge, bei Ärzt:innen würde eine Verfügung zu einer „verfrühten“ Einstellung der Therapie führen. Schließlich befürchteten Patient:innen, durch Patientenverfügung oder Vorsorgevollmacht ihre Zugehörigen unnötig zu belasten.

Die Patient:innen in unserer Studie wiesen ohne Ausnahme eine hohe Symptombelastung durch die Erkrankung und ihre Behandlung auf und hatten ein erhöhtes Risiko, im Krankenhaus zu versterben. Unsere Daten zeigen jedoch, dass diese Patient:innen, die von einer Patientenverfügung oder Vorsorgevollmacht profitieren würden, mehrheitlich keine Verfügung erstellen. Dies bestätigt frühere Untersuchungen [4]. Die Befragung von Patient:innen im hausärztlichen Setting in Nordrhein-Westphalen ergab, dass nur 32 % eine Patientenverfügung erstellt hatten und dass 43 % der Patient:innen die Erstellung vor sich herschob. Der Hauptgrund für die Erstellung war die Sorge um die Einschränkung der eigenen Autonomie [11]. Auch hier war lediglich das Lebensalter ein Prädiktor, weder persönliche Faktoren noch der Gesundheitsstatus zeigten einen Zusammenhang mit der Erstellung. Ähnliche Daten berichten de Heer et al. für intensivmedizinische Patient:innen, bei denen in einer monozentrischen Untersuchung zwischen 2013 und 2014 in insgesamt 39 % der Fälle eine Vorsorgevollmacht und in 29 % eine Patientenverfügungen ermittelt werden konnte [1].

Unsere Daten deuten darauf, dass es Patient:innen häufig an Wissen darüber mangelt, in welcher Situation Verfügungen wichtig wären und wie sie anzufertigen sind. Im Spannungsfeld zwischen Patientenautonomie und Fürsorge der Ärzt:innen beschreibt die nationale Ethikkommission im Bereich der Humanmedizin der Schweiz ein Autoritätsgefälle, da Ärzt:innen meist einen Wissens- und Erfahrungsvorsprung in die Entscheidungssituation einbringen: Deshalb bedeutet *„shared decision-making“* auch, das Verhältnis besser auszubalancieren, indem einerseits die Ärzt:innen patientenorientiert kommunizieren, damit andererseits Patient:innen sich das nötige Wissen aneignen können [15]. Eigene Erfahrungen im Team des palliativmedizinischen Diensts zeigen mehrere Faktoren, die in diesem Zusammenhang wichtig sind. *Erstens* besteht ein Informationsbedarf zur aktuellen Rechtslage. Die Bedeutung einer Vorsorgevollmacht und die Rolle der Patientenverfügung im Behandlungssetting sind vielen Patient:innen nicht hinreichend bekannt. Hier ist ein Informationsbedarf allerdings nicht erst bei Manifestation einer lebenszeitlimitierenden Erkrankung festzustellen, da auch Unfälle und Akuterkrankungen zu einer Einschränkung der kognitiven Fähigkeiten bei Patient:innen führen können. Konkret sollten mögliche Situationen angesprochen werden, in denen diese Verfügungen benutzt werden, z. B. bei der Frage einer Wiederbelebung nach Herzstillstand, mitsamt der inner- und außerklinischen Langzeitergebnisse. Bei der Erstellung einer Vorsorgevollmacht sollten die Patient:innen ihre Wünsche und Sorgen detailliert mit der benannten Person besprechen. Zweitens sind es ganz praktische Aspekte der Umsetzung, die für Patient:innen eine relevante Hürde darstellen. So besteht oft eine Unklarheit darüber, welche Schritte notwendig sind, um eine Vorsorgevollmacht zu erstellen, oder die falsche Annahme, dass diese nur mit einem Notar auszustellen sei. Hier wäre eine bevölkerungsbasierte Aufklärung notwendig, um niederschwellige Beratungsangebote zu vermitteln. Obschon die Beratung im multiprofessionellen Palliativdienst bereits etabliert ist, wurden vor diesem Hintergrund in den Anästhesieambulanzen der Charité entsprechende Dokumente ausgelegt, um allen perioperativen Patient:innen einen niederschwelligen Zugang zur Patientenverfügung und Vorsorgevollmacht zu ermöglichen. Entsprechende Dokumente und Begleitinformationen werden von verschiedenen Quellen zur Verfügung gestellt (beispielsweise von Vereinen – z. B. Home Care Berlin e. V. [https://homecareberlin.de/beratung/] oder dem Bundesministerium für Justiz [siehe https://www.bmj.de/DE/themen/vorsorge_betreuungsrecht/patientenverfuegung/patientenverfuegung_node.html]). Drittens sehen einige Patient:innen keine Notwendigkeit für die Erstellung einer Patientenverfügung oder Vorsorgevollmacht, weil sie davon ausgehen, dass Zugehörige im entsprechenden Fall Präferenzen bereits kennen und a priori befugt sind, diese mit dem Behandlungsteam umzusetzen. Einige Patient:innen berichteten jedoch auch, Schwierigkeiten bei der Verantwortungszuteilung oder den Entscheidungen zu haben, welche medizinischen Maßnahmen sie in bestimmten Situationen wünschen oder ablehnen würden. Insbesondere hier könnte zunächst der Stellenwert der Vorsorgevollmacht besprochen werden, um und dann zunächst eine Werteermittlung durchzuführen. Entsprechende Werteermittlungsbögen werden beispielsweise von der Berliner Home-Care Berlin e. V. zur Verfügung gestellt (siehe https://homecareberlin.de/ueber-uns/downloads/). Häufig spielen Ängste vor dem Lebensende eine Rolle oder führen zu Konflikten in der Familie, sodass die Erstellung einer Verfügung aufgeschoben wird.

Schlussendlich zeigten sich explizite oder implizite religiöse Vorstellungen von Relevanz, da sie zu unterschiedlichen Bewertungen von therapeutischen Maßnahmen führen können. Entsprechende Einordnungen thematisierte Fred Salomon aus der Sicht verschiedener Religionen zu Therapiezieländerungen, wobei eine große Heterogenität innerhalb und außerhalb verschiedener Weltanschauungen deutlich wurde [10].

Insgesamt besteht damit ein durchaus zeitintensiver Beratungsbedarf bei Patient:innen. Nicht selten benötigen sie mehrere Gespräche mit ärztlichen und pflegerischen Mitbehandlern oder Vertretern anderer Professionen, z. B. Sozialdienstmitarbeiter:innen, für diesen Prozess. Ein zentraler Gegenstand der Versorgung von Patient:innen, insbesondere mit einer lebensbegrenzenden Erkrankung, ist es deshalb, aktiv zum Thema Vorsorgevollmacht und Patientenverfügung zu beraten, um bestehende Barrieren möglichst auszuräumen und sicherzustellen, dass Patient:innen ihre individuellen Wünsche und Bedürfnisse formulieren können.

Die hier vorliegende Studie weist eine Reihe von Limitationen auf. Neben dem monozentrischen Studiensetting war die Auswahl der Patient:innen durch das Zuweisungsverhalten der behandelnden Ärzt:innen bestimmt und daher nicht repräsentativ. Patient:innen ohne notwendige Sprach- oder Kommunikationskompetenz und intensivmedizinische Patient:innen mit Sedierung oder Intubation wurden nicht eingeschlossen, obwohl Vorausverfügungen für diese Gruppen besonders nützlich wären [5]. Eine Stärke der Studie ist die Größe der Stichprobe. Unsere Studie erfasste nicht, wie viele Patient:innen nach entsprechender Beratung eine Vorsorgevollmacht und Patientenverfügung anfertigten. Da unser Beratungsprozess auch teilweise Zugehörige einschloss, zog sich die tatsächliche Erstellung oft bis in die ambulante Weiterversorgung und war deshalb nicht Teil der durchgeführten Erhebung.

## Ausblick

Der Bedarf an Beratung zu Patientenverfügungen und Vorsorgevollmachten bei Patient:innen mit einer hohen Krankheitslast und einem hohen Risiko zu versterben ist hoch. Insbesondere benötigen die Patient:innen und ihre Zugehörigen mehr Informationen darüber, in welchen klinischen Situationen diese Verfügungen benötigt werden und welche Schritte zur Erstellung nötig sind. Aufgrund der Komplexität der möglichen zukünftigen Entscheidungssituationen benötigen viele Patient:innen Unterstützung bei der Entwicklung ihrer Präferenzen. Hierfür sind meist längere Beratungsprozesse nötig. Der palliativmedizinische Konsildienst im Krankenhaus mit seinem multidisziplinären Team ist geeignet, solche Beratungen durchzuführen. Dabei dürfte aufgrund der demografischen Entwicklung und zunehmender Morbidität die Notwendigkeit entsprechender niederschwelliger Angebote der Vorsorge in vielen Fachgebieten bestehen. Informationsmaterial in verschiedenen Ambulanzen könnte ergänzt werden durch aktive Angebote vor und nach einem Krankenhausaufenthalt.
